# Supplementary figures and images for: Characterizing the Pathogenesis and Immune Response of Equine Herpesvirus 8 Infection in Lung of Mice
Source: Animals (Basel). 2022 Sep 20;12(19):2495. doi: 10.3390/ani12192495 (PMC9559255; doi:10.3390/ani12192495)

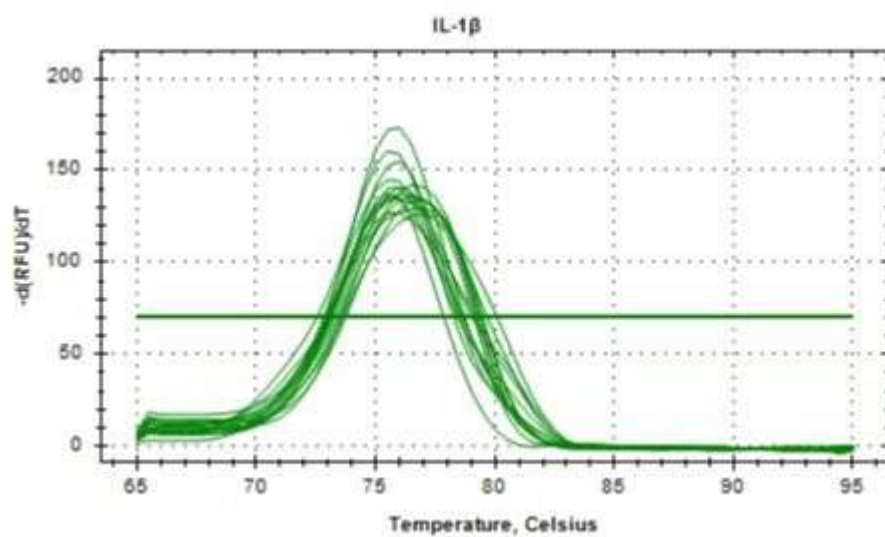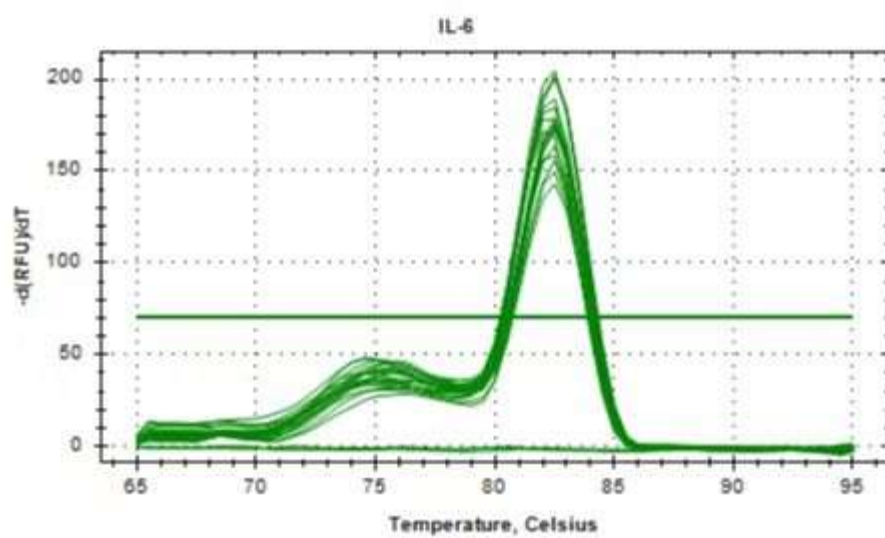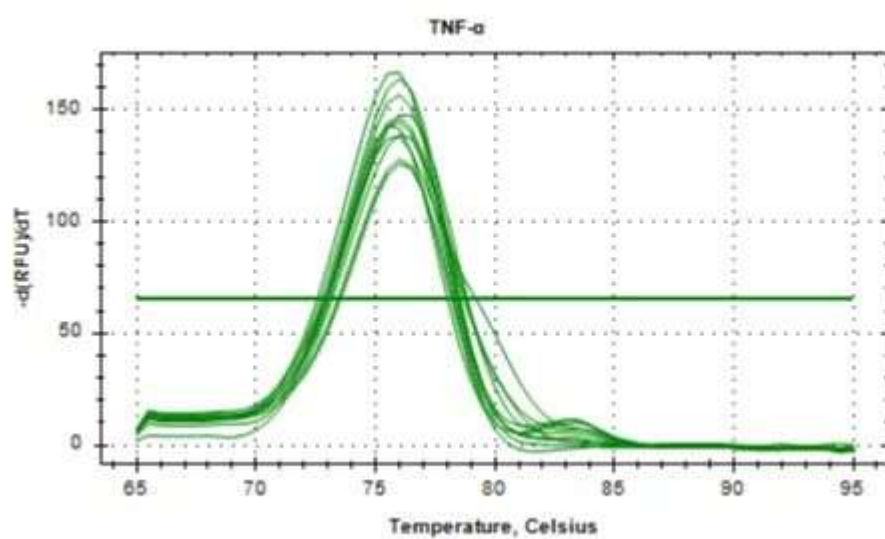

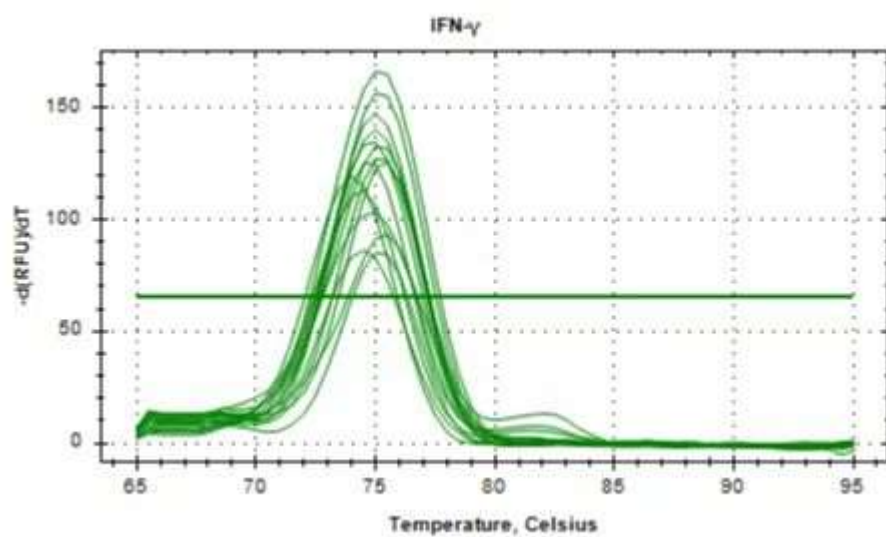

Supplement: Supplementary file 1 [file animals-12-02495-s001.zip › animals-1895450-supplementary.pdf]
